# Current trend in treatment of glioblastoma in Japan: a national survey using the diagnostic procedure combination database (J-ASPECT study-glioblastoma)

**DOI:** 10.1007/s10147-021-01929-5

**Published:** 2021-05-11

**Authors:** Yusuke Funakoshi, Nobuhiro Hata, Daisuke Kuga, Ryusuke Hatae, Yuhei Sangatsuda, Yutaka Fujioka, Kosuke Takigawa, Koji Yoshimoto, Masahiro Mizoguchi, Koji Iihara

**Affiliations:** 1 Department of Neurosurgery, Graduate School of Medical Sciences, Kyushu University, 3-1-1 Maidashi, Higashi-Ku, Fukuoka, 812-8582 Japan; 2 Department of Neurosurgery, Graduate School of Medical and Dental Sciences, Kagoshima University, 8-35-1 Sakuragaoka, Kagoshima, 890-8520 Japan; 3 Department of Neurosurgery, National Cerebral and Cardiovascular Center, 6-1 Kishibe-Shimmachi, Suita, Osaka 564-8565 Japan

**Keywords:** Glioblastoma, Temozolomide, Bevacizumab, Diagnostic procedure combination, Treatment centralization

## Abstract

**Background:**

In the treatment for glioblastoma (GBM), treatment modalities, such as bevacizumab (BEV) and carmustine wafers implants have been approved in Japan since 2013. However, it is unclear whether such a trend in treatment complexity can accelerate treatment centralization. The aim of this study was to reveal the current trend in the treatment of GBM in Japan.

**Methods:**

We used diagnostic procedure combination (DPC) database to analyze the data of 1,774 patients from 305 institutions between April 2016 and March 2019. To analyze the situations associated with first-line BEV use during concurrent TMZ (temozolomide)-radiotherapy, we compared TMZ alone and TMZ–BEV groups.

**Results:**

Of the 1,774 patients with GBM, tumor removal by craniotomy was performed in 1,572 (88.6%) patients, and stereotactic biopsy was performed in 156 (8.8%) patients. A total of 1,229 (69.3%) patients underwent radiotherapy, and 1,287 (72.5%) patients underwent chemotherapy. TMZ alone was administered to 878 (68.2%) and TMZ combined with BEV in 381 (29.6%) patients. In the TMZ–BEV group, as compared to the TMZ-alone group, the rate of discharge to home was significantly lower (*P* = 0.0044), and the rate of stereotactic biopsy was significantly higher (*P* < 0.0001). No significant difference was observed in the distribution of patients between the TMZ alone and TMZ–BEV groups depending on the scale of institution (*P* = 0.1240).

**Conclusion:**

First-line BEV administration seems to be selected properly regardless of the institutional scale. This Japan-wide study of GBM treatment revealed that high level and newly introduced treatments have been steadily generalized in Japanese institutions.

## Introduction

Glioblastoma (GBM) is the most common primary brain tumor and is well known to have one of the most dismal prognoses in cancer. The conventional standard treatment of GBM consists of maximal feasible resection, followed by radiotherapy (RT) and chemotherapy (CTX) with temozolomide (TMZ). Because adverse events of TMZ are relatively mild, and it is administered orally, this treatment was widely generalized in a routine inpatient hospital environment. Since 2013, however, bevacizumab (BEV) has been approved in Japan as an insurance-covered first-line drug for GBM because of the improvement in progression-free survival reported in two randomized clinical trials, AVAglio and RTOG 0825 [1, 2]. Furthermore, carmustine wafers implants have also been approved as local CTX administration for high-grade glioma because of their favorable outcomes [3–6]. In addition, intraoperative support, such as navigation, has recently become more important. Thus, an increase in the treatment expertise due to diversification of treatment modalities may cause progression in treatment centralization. However, few studies have analyzed in detail where and how GBM was treated in Japan.

In 2002, the Japanese government introduced a per diem prospective payment system with a diagnosis-related group-like grouping, which is called diagnostic procedure combination (DPC) [7]. The data for practices can be obtained from DPC, and an attending physician is responsible for clinical data entry for each patient. We have previously reported the discharge outcomes in cerebrovascular disease patients in a nationwide retrospective analysis using the DPC database (J-ASPECT study) [8–12]. In addition, fields other than cerebrovascular diseases, such as traumatic brain injury (J-ASPECT study-traumatic brain injury) have also been reported recently [13].

Using the DPC database, we have previously reported current trends and healthcare resource usage in the treatment of primary malignant brain tumors in Japan (J-ASPECT study-brain tumor) [14]. Although in the previous study, the most serious limitation was the lack of information on various histologically different tumors, detailed diagnosis codes have been available since 2016, and only cases diagnosed with GBM could be extracted. The aim of this study was to reveal the current trend in the GBM treatment in Japan to improve and standardize the management of this disease.

## Materials and methods

### Data acquisition

**Fig. 1:**
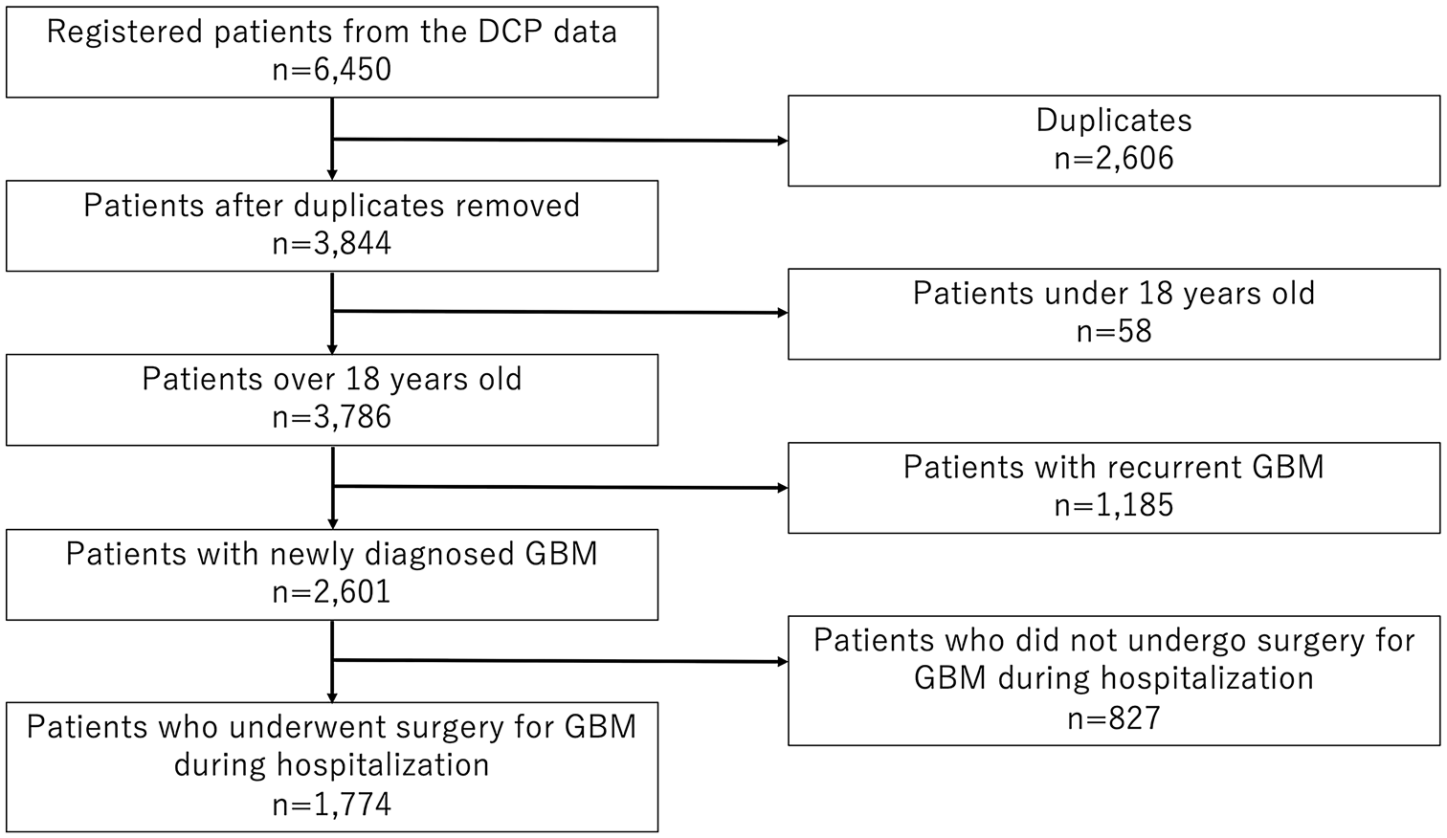
Flow chart of the included patients with glioblastoma (GBM) in the present study

We obtained the DPC data of 6,450 patients from 471 institutions between April 2016 and March 2019. Duplicates, patients under 18 years old, those with recurrent GBM, and those without surgery for GBM were excluded, and eventually, the DPC data of 1,774 patients from 305 institutions were analyzed (Fig. 1). Discharge destination, status at discharge, length of stay, and medical costs were evaluated. Moreover, treatment modalities, including surgery, RT, and CTX regimens, were also evaluated. The DPC reimbursement system paid for the surgery, RT, and CTX as fee-for-service, while other costs for hospitalization, medications, blood examination, imaging examination, and physician time were inclusive. As the cost of chemotherapeutic drugs was inclusive, the dose and duration of CTX were not reflected in the DPC reimbursement. The medical cost was converted into US dollars based on an exchange rate of 104 Japanese yen per US dollar.

### Treatment modalities

In the DPC, surgical procedures and adjuvant therapy were specified by subcodes. Demographic data of GBM were stratified according to the treatment modalities described below.

### Surgery

Surgical treatments were classified and specified using K-code. The data on surgical procedures directly related to tumor treatment were extracted. As K-codes for representative surgery, code K169 (tumor removal by craniotomy) was used to extract data for patients who underwent removal of the tumor by craniotomy, and code K154-3 (stereotactic biopsy) was used to extract data for patients who underwent stereotactic biopsy. As K-codes for minor surgery, code K148 (craniotomy only), K145 (extraventricular drainage), K174 (operation for hydrocephalus), K151-2 (tumor removal by extended skull base craniotomy), K171 (transsphenoidal tumor removal), K164 (hematoma removal by craniotomy), and K191 (spinal tumor removal) were included. In patients who underwent more than two surgeries, tumor removal by craniotomy after stereotactic biopsy was counted as tumor removal by craniotomy, and operation for hydrocephalus after extraventricular drainage was counted as operation for hydrocephalus. In addition, intraoperative support, including navigation, 5-aminolevulinic acid (ALA), electrophysiological monitoring, and awake surgery were evaluated. Because carmustine wafer implants were approved in 2013, the status of carmustine wafer usage was also evaluated.

### Radiotherapy

RT was classified and coded according to the treatment modality. External beam therapy (EBT) was coded as M001, intensity-modulated radiation therapy (IMRT), stereotactic radiosurgery (SRS), and stereotactic RT (SRT) were coded as M001-IMRT, M001-2, and M001-3, respectively. In this study, we identified patients treated with RT and subcategorized them based on the modality using these codes.

### Chemotherapy

TMZ and BEV were the representative chemotherapeutic drugs used for GBM treatment. Other insurance-adapted drugs for GBM were nimustine (ACNU), interferon, methotrexate, ifosfamide, cisplatin, carboplatin, vincristine, cyclophosphamide, and procarbazine. We considered that CTX had been administered when there was a code for CTX, and one of these drugs was listed.

### First-line bevacizumab

Because the unique BEV usage regardless of the clinical stage has been approved in Japan, first-line BEV has been administered to patients with severe clinical conditions to improve their performance status. In this study, first-line BEV was defined as BEV usage during concomitant TMZ and RT during hospitalization. To evaluate the efficacy of additional BEV, we compared with the outcomes between the groups treated with concomitant TMZ alone and RT (TMZ-alone group), and additional BEV during concomitant TMZ and RT (TMZ–BEV group). In this analysis, patients who underwent more than 3 weeks of concomitant TMZ and RT were included.

### Statistical analyses

Statistical analyses were performed using statistical software (JMP software, version 15; SAS Institute). To compare between the TMZ alone and TMZ–BEV groups, sex, discharge destination, status at discharge, type of surgery, and difference between institutions and areas were evaluated using the chi-square test; while age, length of stay, and medical cost were evaluated using the Mann–Whitney *U* test. *P* < 0.05 was considered to indicate statistically significant difference.

### Ethical statement

The present study was approved by the Institutional Review Board of Kyushu University, which waived the requirement for informed consent from the participants. The study was conducted in accordance with the 1964 Declaration of Helsinki (as revised in Fortaleza, Brazil, October 2013).

## Results

### Clinical characteristics and outcomes

Table 1 summarizes the clinical characteristics and outcomes in the patients included in this study. Of the 1,774 patients with GBM, 56.9% were men and 43.1% were women. The median age was 68.0 years. The majority were referral patients (77.6%), and emergency transportation accounted for 15.1%. The discharge destination was home in 1,148 (64.7%) patients and other hospitals in 515 (29.0%). Death during hospitalization was recorded for 61 (3.4%) patients. Good outcome at discharge was achieved in 1,497 (84.4%) patients, and the outcome at discharge was poor in 81 (4.6%) patients. The median length of stay was 66.0 days, and the median medical cost was 58,335 dollars.

**Table 1:** Clinical patient characteristics and outcomes

Characteristics	
Total number	1774
Age (years)	68.0 (59.0–76.0)
Sex	
Male	1009 (56.9%)
Female	765 (43.1%)
Trends of consultation	
Referral	1377 (77.6%)
Transfer	118 (6.7%)
Emergency transportation	268 (15.1%)

Outcomes	
Discharge destination	
Home	1148 (64.7%)
Care facility	43 (2.4%)
Other hospital	515 (29.0%)
Death	61 (3.4%)
Other	7 (0.4%)
Status at discharge	
Good	1497 (84.4%)
No change	157 (8.9%)
Poor	81 (4.6%)
Other	39 (2.2%)
Length of stay (days)	66.0 (40.0–80.3)
Medical cost (thousand dollars)	58.3 (39.7–72.1)

The data for age, length of stay, and medical cost are presented as median (interquartile range)

### Treatment modalities

**Fig. 2:**
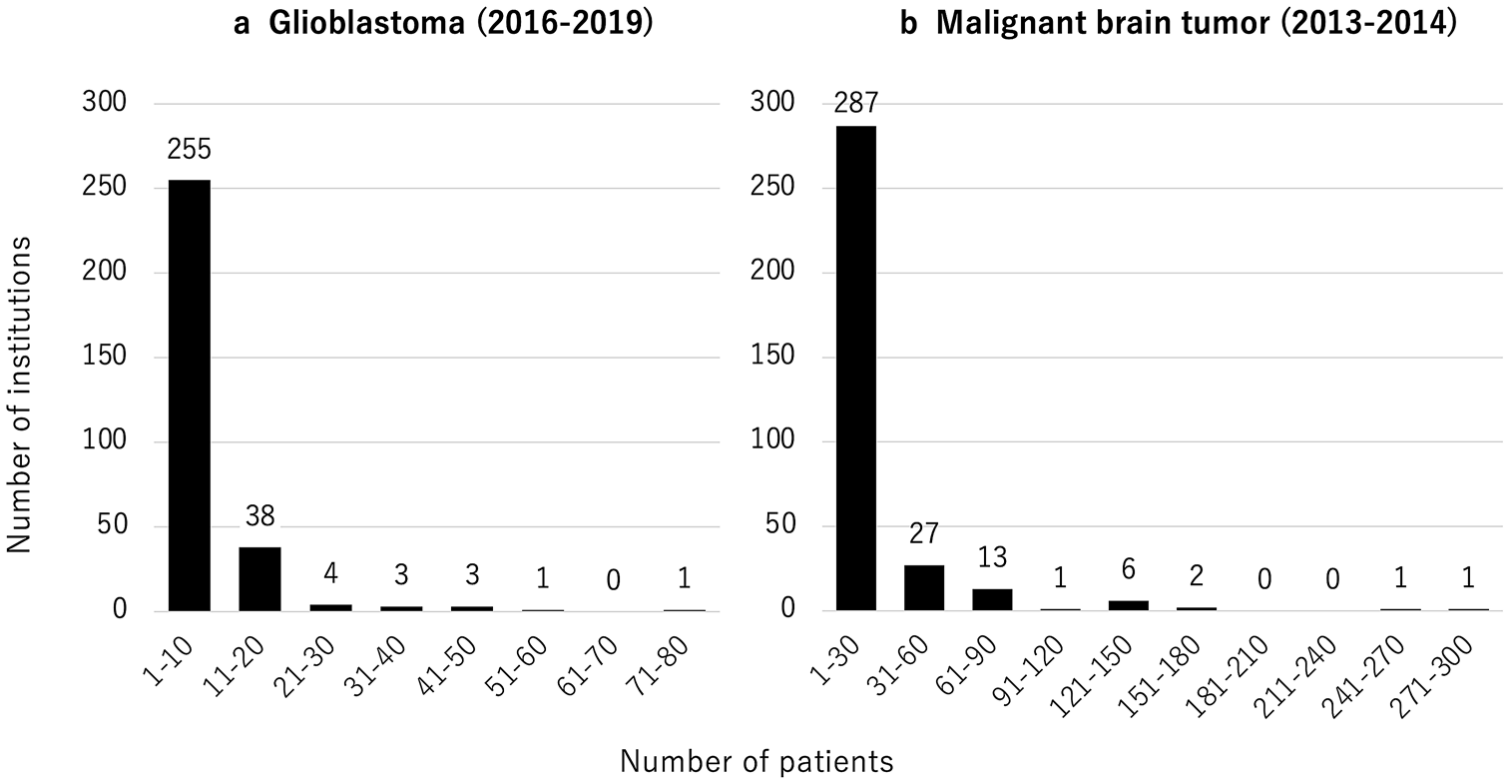
**a** The distribution of the number of patients with GBM who underwent surgery in each institution between 2016 and 2019 (J-ASPECT study-Glioblastoma). **b** The distribution of the number of patients with primary malignant brain tumor in each institution between 2013 and 2014 (J-ASPECT study-Brain Tumor)

**Fig. 3:**
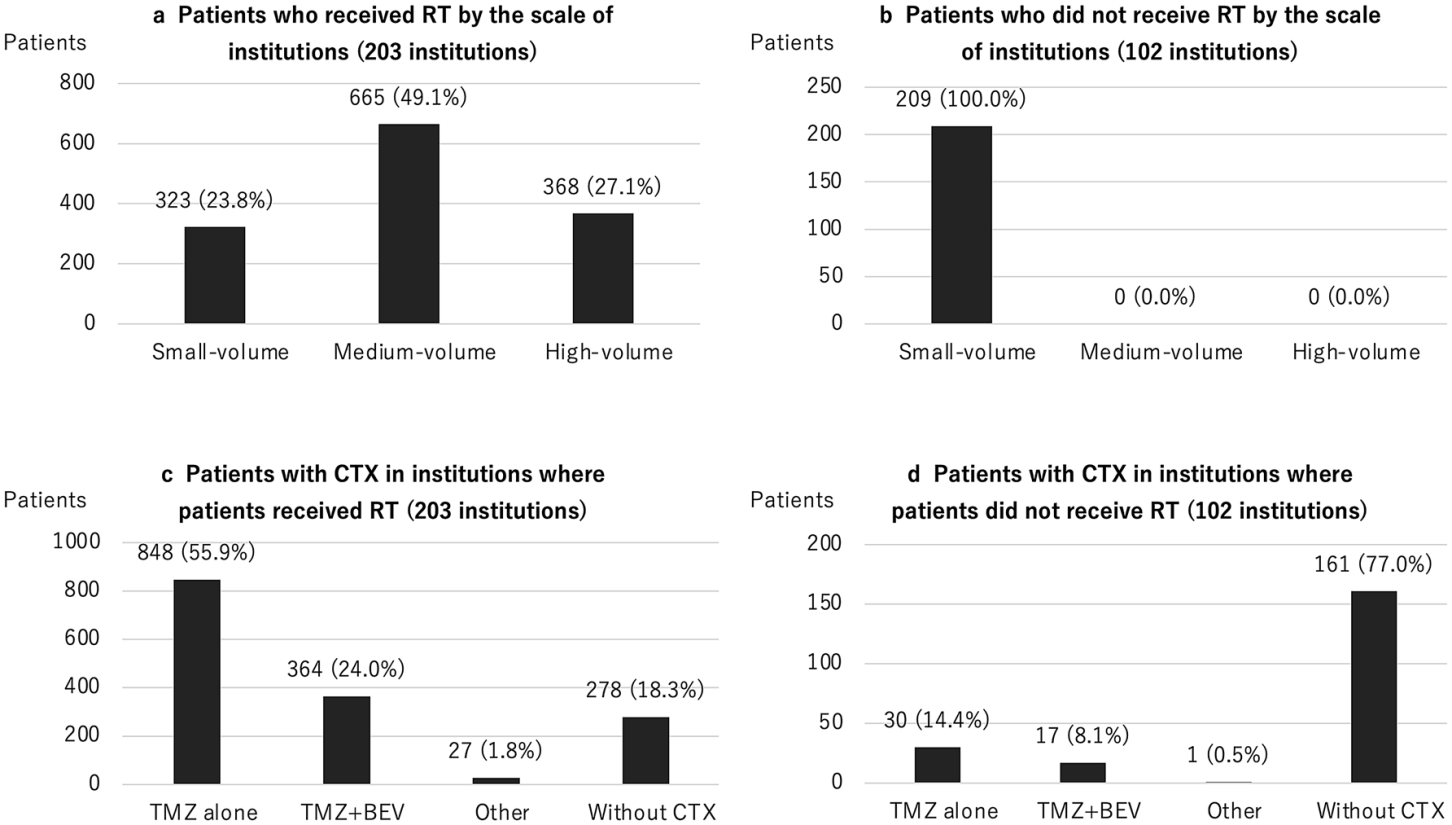
**a**, **b** The distribution of the number of patients with GBM treated with and without RT among institutions by the scale of the institutions. **c**, **d** The distribution of the number of patients with GBM treated with CTX in institutions where the patients did and did not receive RT. *CTX* chemotherapy, *RT* radiotherapy, *TMZ* temozolomide, *BEV* Bevacizumab

**Fig. 4:**
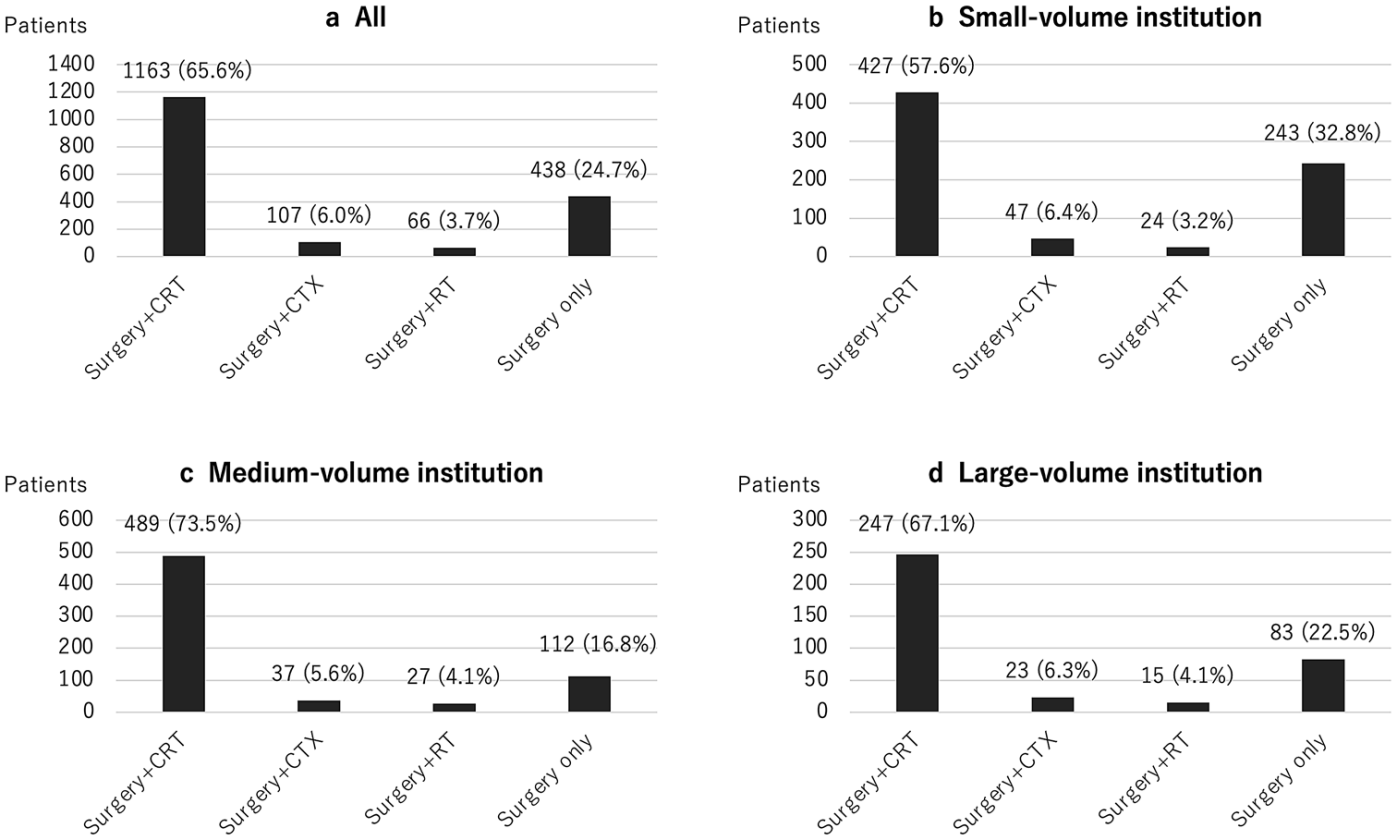
**a**–**d** The distribution of the number of patients with GBM treated with different treatment modalities depending on the scale of the institution. *CRT* chemoradiotherapy, *CTX* chemotherapy, *RT* radiotherapy

Table 2 summarizes the treatment modalities for GBM. Tumor removal by craniotomy was performed in 1,572 (88.6%) patients, and stereotactic biopsy was performed in 156 (8.8%) patients. As intraoperative support, navigation was used in 1,303 (73.4%) patients, and 5-ALA was used in 1,032 (58.2%) patients. Intraoperative electrophysiological monitoring was often used; in particular, motor evoked potential (MEP) monitoring was used in 797 (44.9%) patients. Awake surgery was performed in 29 patients (1.6%). A carmustine wafer implant, which is generally an option for patients in whom subtotal removal or more was achieved, was used in 551 (31.1%) patients. The number of patients registered in a single institution between April 2016 and March 2019 varied between 1 and 74, with a median of 3 (Fig. 2a), indicating that many institutions performed surgery for less than 10 patients with GBM for 3 years (small-volume institution), and a few performed for more than 30 patients (high-volume institution). This trend was similar to our previous report, which summarized malignant brain tumors between April 2013 and March 2014 (Fig. 2b) [14]. A total of 1,229 (69.3%) patients with GBM underwent RT at the first hospitalization. Among these patients, the patients who underwent multiple types of RT during one hospitalization were included; EBT was performed in 949 (77.2%), IMRT in 362 (29.5%), SRS in 2 (0.2%), and SRT in 6 (0.5%) patients. A total of 1,287 (72.5%) patients underwent CTX during the first hospitalization. Among these patients, TMZ alone was administered to 878 (68.2%), TMZ combined with BEV to 381 (29.6%), ACNU alone to 6 (0.5%), and other chemotherapeutic drugs to 22 (1.7%) patients. Of the 305 registered institutions, 102 did not have patients who were treated with RT. All of these institutions were small-volume institutions, and 77.0% of patients in these institutions did not undergo CTX (Fig. 3). Figure 4 demonstrates the distribution of the number of patients treated with different treatment modalities depending on the scale of the institution. In small-volume institutions, the rate of surgery was higher than that in medium- and large-volume institutions.

**Table 2:** Treatment modalities

Surgery	
Tumor removal by craniotomy	1572 (88.6%)
Stereotactic biopsy	156 (8.8%)
Other	46 (2.6%)
Intraoperative support	
Navigation	1303 (73.4%)
5-ALA	1032 (58.2%)
MEP	797 (44.9%)
SEP	72 (4.1%)
VEP	3 (0.2%)
Awake surgery	29 (1.6%)
Local CTX	
Carmustine wafers	551 (31.1%)

RT (*n* = 1229)	
External beam therapy	949 (77.2%)
IMRT	362 (29.5%)
SRS	2 (0.2%)
SRT	6 (0.5%)

Systematic CTX (*n* = 1287)	
TMZ alone	878 (68.2%)
TMZ + BEV	381 (29.6%)
ACNU alone	6 (0.5%)
Other	22 (1.7%)

5-*ALA* 5-aminolevulinic acid, *MEP* motor evoked potential, *SEP* somatosensory, *VEP* visual evoked potential, *CTX* chemotherapy, *RT* radiotherapy, *IMRT* intensity modulated radiation therapy, *SRS* stereotactic radiosurgery, *SRT* stereotactic radiotherapy, *TMZ* temozolomide, *BEV* bevacizumab, *ACNU* nimustine

### Comparison between the TMZ alone and the TMZ–BEV groups

There were 708 and 275 patients in the TMZ alone and the TMZ–BEV groups, respectively (Table 3). There was no significant difference in age (*P* = 0.4169) between the groups, but men were more frequently treated with TMZ alone (*P* = 0.0321). The rate of transfer to other hospitals was higher in the TMZ–BEV group compared to the TMZ-alone group (TMZ alone: 23.7%, TMZ–BEV: 30.9%), and the rate of discharge to home was lower (TMZ alone: 71.6%, TMZ–BEV: 60.7%). There was a significant difference in discharge destination (*P* = 0.0044) between the groups. In addition, the rate of good outcome was significantly lower (TMZ alone: 89.2%, TMZ–BEV: 82.1%, *P* = 0.0135) and the rate of stereotactic biopsy was significantly higher (TMZ alone: 4.5%, TMZ–BEV: 16.0%, *P* < 0.0001) in the TMZ–BEV group, indicating that patients with severe conditions, such as unresectable tumors and poor performance, were treated with first-line BEV. The length of stay was significantly longer in the TMZ–BEV group (median length of stay in TMZ-alone group: 73.0 days, TMZ–BEV group: 78.0 days, *P* = 0.0002), and medical costs were significantly higher in the TMZ–BEV group (TMZ-alone group: 65,665 dollars, TMZ–BEV group: 76,157 dollars, *P* < 0.0001). No significant difference was observed in the distribution of patients between the TMZ alone and the TMZ–BEV groups depending on the scale of institution (*P* = 0.1240).

**Table 3:** Comparison between the TMZ alone and the TMZ–BEV groups

	TMZ alone group (*n* = 708)	TMZ-BEV group (*n* = 275)	*P* value
Age (years)	67.0 (58.0–73.0)	68.0 (58.0–74.0)	0.4169
Men	424 (59.9%)	144 (52.4%)	0.0321*
Discharge destination			0.0044*
Home	507 (71.6%)	167 (60.7%)	
Care facility	16 (2.3%)	14 (5.1%)	
Other hospital	168 (23.7%)	85 (30.9%)	
Death	15 (2.1%)	6 (2.2%)	
Other	2 (0.3%)	3 (1.1%)	
Status at discharge			0.0135*
Good	632 (89.2%)	226 (82.1%)	
No change	48 (6.8%)	31 (11.3%)	
Poor	19 (2.7%)	9 (3.3%)	
Other	9 (1.3%)	9 (3.3%)	
Surgery			< 0.0001*
Tumor removal by craniotomy	668 (94.4%)	229 (83.3%)	
Stereotactic biopsy	32 (4.5%)	44 (16.0%)	
Other	8 (1.1%)	2 (0.7%)	
Length of stay (days)	73.0 (65.0–84.0)	78.0 (68.0–91.0)	0.0002*
Medical cost (thousand dollars)	65.7 (57.8–74.1)	76.2 (63.6–85.8)	< 0.0001*
Institution			0.1240
Small volume	277 (39.1%)	89 (32.4%)	
Medium volume	280 (39.6%)	125 (45.4%)	
High volume	151 (21.3%)	61 (22.2%)	

The data for age, length of stay, and medical cost are presented as median (interquartile range)

*BEV* bevacizumab, *TMZ* temozolomide

*Indicates statistical significance

## Discussion

An increasing interest in evidence-based medicine and improving the quality of patient care and patient safety has created a demand for accurate and accessible information on activity and trends in clinical practice worldwide [15]. In the J-ASPECT study, we have analyzed clinical practice in Japan using a nationwide DPC database to visualize real-world clinical practice, promote science, and improve the quality of patient care. In addition, the Japan Neurosurgical Society started the Japan Neurosurgical Database (JND), a nationwide, hospital-based multicenter registry in 2016, and analysis of real-world neurosurgical practice in Japan is expected to develop further in the near future [16]. In this study, we analyzed the current trend of GBM treatment using the DPC database in Japan. The advantage of using the DPC database is that patient and hospital information, diagnosis, procedures, and administrative claim data are completely enumerated for all patients in the participating hospitals [14]. We hypothesized that an increase in the treatment complexity due to diversification of treatment modalities can lead to acceleration of treatment centralization. In our study, we demonstrated the actual usage of various treatment options for GBM in Japan using the DPC database. However, the number of surgeries for GBM depending on the scale of institution between April 2016 and March 2019 was similar to that shown in our previous report, which summarized malignant brain tumors between April 2013 and March 2014, indicating that the treatment centralization has not progressed since 2013. High level and newly introduced treatments have been well generalized for patients with GBM in various institutions.

In this study, most patients underwent tumor removal by either craniotomy (88.6%) or stereotactic biopsy (8.8%). This data seem to indicate that Japanese neurosurgeons tend to make efforts to achieve feasible maximal resections in most situations; however, the possibility of overestimation should be taken into consideration because the data about the extent of resection is not included in the DPC database; therefore, a minimal partial removal aiming to biopsy could be also registered as tumor removal by craniotomy. Intraoperative supports, such as navigation, 5-ALA fluorescence, and MEP monitoring, were used in 1,303 (73.4%), 1,032 (58.2%), and 797 (44.9%) patients, respectively, suggesting that these operative procedures have reached generalized options in the majority of neurosurgical institutions. The usage rate of carmustine wafer implants was over 30%, suggesting that this procedure has been also well generalized, taking into consideration that carmustine wafers implants were not often available for emergency surgery and in institutions where pathological diagnosis during surgery was not available, and was generally recognized as an option for patients in whom subtotal removal or more was achieved. Despite the wide usage of carmustine wafers implants in Japan, there has been only one subgroup analysis in a randomized controlled trial in which carmustine wafers implants have shown to prolong survival in GBM [6, 17]. Owing to insufficient evidence about their efficacy, carmustine wafers implants have not been utilized as standard treatment. To resolve the issue, JCOG1703, which is a phase III randomized controlled trial for carmustine wafers implants with the add-on Stupp regimen [18] has been ongoing in Japan.

Because the first-line BEV is approved only in Japan, Japanese data are precious to evaluate the efficacy of such unique BEV usage. Since BEV approval in 2013, Japanese real-world data of first-line BEV have been accumulated. Although these data were derived from retrospective studies in Japanese institutions, the positive impact of first-line BEV on the overall survival prolongation has been implied. These reports enhanced the advantages of first-line BEV for severe clinical conditions, such as unresectable tumors and poor performance status [19–22]. In this study, although the timing of administration of BEV depended on each institution, there were 275 patients in the TMZ–BEV group as compared to 708 in the TMZ-alone group, indicating that first-line BEV was administered in nearly 30% of patients in Japanese institutions. In the TMZ–BEV group, the rate of patients with poor outcome, who were not able to be discharged to home, was significantly higher, suggesting that first-line BEV tended to be administrated as a treatment option for patients with severe clinical conditions. In addition, first-line BEV was used more frequently in patients with stereotactic biopsy. This outcome also revealed that BEV was often used in patients with unresectable tumors. Taken together, first-line BEV seems to be used for the purpose of maintaining performance status during concurrent chemoradiotherapy, as we have suggested previously [21, 22], and the selective usage of BEV for patients undergoing stereotactic biopsy justified the usage of first-line BEV. However, we should take into consideration the increase in the length of stay and medical cost in patients who are treated with TMZ combined with BEV. Although we suggest BEV for GBM patients with severe clinical conditions, BEV should be appropriately used for eligible cases because of prolongation of length of stay and high medical cost. However, the outcome in the present study may indicate that patients who were hospitalized for a long time had more chances to use BEV. BEV can contribute to the improvement of performance status [21], which should prevent long-term hospitalization. Accordingly, long-term medical costs should be reduced by BEV administration at the appropriate timing.

Differences in treatment methods depending on the scale of institution were among other issues to be addressed. In this study, of 305 registered institutions, 102 small-volume institutions did not have patients treated with RT, and 77.0% of patients in these institutions did not undergo CTX. This outcome suggested that, in some small-volume institutions, only surgery was performed, and adjuvant therapy, such as RT and CTX, was administered after the transfer to another high-volume institution. Because of the complexity of diagnosis with the introduction of molecular diagnosis and diversification of treatment modalities, the current standard multimodal treatment for patients with GBM seems to be not manageable in most small-volume institutions. However, in the comparison between the TMZ alone and TMZ–BEV groups, no significant difference was observed in the distribution of patients depending on the scale of institution. This outcome also suggested that high level and newly introduced treatments have been widely performed for patients with GBM in various institutions. To resolve this contradiction, further analysis of population-based studies, such as JND, is expected.

There are several limitations in this study. First, detailed information concerning the performance status of patients, genetic markers, tumor size, and extent of resection was not available in the DPC database. Second, the DPC database only includes mortality data for the corresponding admission period, and information on patient outcomes after discharge was not included. Because the survival rate could not be calculated, and outpatient data were not included, the efficacy of the treatments in the survival of patients with GBM (e.g., first-line BEV, carmustine wafers implants, IMRT) could not be analyzed. The DPC database only revealed the type of treatment; therefore, there is a need for further investigations combining the DPC database with other data after discharge to assess survival data. Third, the data regarding medical costs should be carefully interrupted. Although the medical costs of the patients treated with TMZ combined with BEV were higher than those of the patients treated with TMZ alone, the breakdown of the medical costs was not available in the DPC database. The medical costs might have increased due to BEV usage and long-term hospitalization. However, the tumor removal was more expensive than stereotactic biopsy, which was frequently performed in the patients treated with TMZ combined with BEV. Recently, elderly patients, who are more likely to be administered BEV at an early stage, have been treated with short course radiotherapy [23], which is cheaper than a conventional one. We should consider these factors to strictly analyze the medical costs. Furthermore, in the DPC database system, the attending physician is responsible for inputting all clinical data for each patient, so there is a potential risk of inaccurate data. In addition, the outcomes in this study may not strictly reflect the current state of treatment in Japan, because not all institutions in Japan were registered in the J-ASPECT study. Despite several limitations, up-to-date information has been accumulated in the DPC database, and nationwide studies using the DPC database could provide new evidence for clinical practice from a general population reflecting real-world conditions.
